# Inhibition of Increased Circulating Tfh Cell by Anti-CD20 Monoclonal Antibody in Patients with Type 1 Diabetes

**DOI:** 10.1371/journal.pone.0079858

**Published:** 2013-11-20

**Authors:** Xinyu Xu, Yun Shi, Yun Cai, Qingqing Zhang, Fan Yang, Heng Chen, Yong Gu, Mei Zhang, Liping. Yu, Tao Yang

**Affiliations:** 1 Department of Endocrinology, The First Affiliated Hospital of Nanjing Medical University, Nanjing, Jiangsu Province, China; 2 Barbara Davis Center for Childhood Diabetes, University of Colorado Denver, Aurora, Colorado, United States of America; University of Hong Kong, China

## Abstract

**Objectives:**

Follicular helper T (Tfh) cells exert an important role in autoimmune diseases. Whether it might be involved in type 1 diabetes (T1D) is unknown. Our aim was to investigate the role of Tfh cells in patients with T1D and the effect of anti-CD20 monoclonal antibody (rituximab) on Tfh cells from T1D patients.

**Patients and Methods:**

Fifty-four patients with T1D and 37 healthy controls were enrolled in the current study. 20 of those patients were treated with rituximab. The frequencies of circulating CD4^+^CXCR5^+^ICOS^+^T cells were analyzed by flow cytometry. The serum autoantibodies were detected by radioligand assay. The levels of IL-21, IL-6 and BCL-6 were assessed using ELISA and/or real-time PCR.

**Results:**

Increased frequencies of circulating Tfh cells together with enhanced expression of IL-21 were detected in patients. The correlation between the frequencies of circulating Tfh cells and the serum autoantibodies or C-peptide level was comfirmed. After rituximab therapy, follow-up analysis demonstrated that the frequencies of circulating Tfh cell and serum IA2A were decreased. The levels of IL-21, IL-6 and Bcl-6 mRNA were decreased after treatment. Furthermore, beta cell function in 10 of 20 patients was improved.

**Conclusions:**

These data indicate Tfh cells may participate in the T1D-relatede immune responses and B cells might play a role in the development of Tfh responses in the disease progression.

## Introduction

Type 1 diabetes (T1D) is a disease resulting from the specific destruction of beta cells within pancreatic islets by autoreactive CD4^+^ and CD8^+^ T cells. Although T cells are dominant determinants of beta-cell destruction in NOD mice and humans, B cells and humoral immunity may also play a role in T1D development or disease progression [1]. B cells infiltrate the pancreatic islets of NOD mice during the autoimmune response that precedes the onset of type 1 diabetes [2]. They may contribute to diabetes in NOD mice by supporting development of tertiary lymphoid structures in the vicinity of pancreatic islets where pathogenic T cells might be activated [3]. NOD mice rendered deficient in B cells, either by treatment with B cell-depleting antibodies or through the introduction of an immunoglobulin (Ig) μ chain gene knockout (NOD.*Igμnull* mice), were found to be highly resistant to T1D [4], [5]. Recently, the importance of B cells in Type 1 diabetes has been resurrected based on the clinical efficacy of B cell depletion with anti-CD20 (rituximab) in T1D patients [6].

Circulating autoantibodies to islet antigens are also strongly associated with development of the disease [7]. Once helper T cells are activated, they will induce B cells to secrete autoantibodies to autoantigens expressed in the pancreatic beta cells. Autoantibodies to insulin(IAA), the tyrosine- phosphatase-like protein IA-2, the 65-KD form of glutamate decarboxylase (GAD65) and zinc transporter 8 (ZnT8) autoantibodies are routinely used in the evaluation of the autoimmune response, risk assessment of individuals and progression to type 1 diabetes [8]. However, indirect evidence against a pathogenic role for autoantibodies came from the decreased incidence of type 1 diabetes in offspring of diabetic mothers compared with diabetic fathers, despite transmission of maternal anti-islet autoantibodies [9]. Recently, Silva et al [10] had investigated that the autoantibodies were potent cofactors in type 1 diabetes progression. This observation could indicate that the effects of anti-islet antibodies are influenced by underlying heterogeneity in the efficiency of CD4^+^ T cell tolerance mechanisms, which are affected by variability in MHC II antigen presentation.

In recent years, T follicular helper (Tfh) cells have emerged as the subpopulation of CD4^+^ T cells required for the formation of germinal centers (GCs) and provision of help to B cells [11]–[13]. Expression of CXCR5, together with loss of the T cell zone–homing chemokine receptor CCR7, allows Tfh cells to relocate from the T cell zone to the B cell follicles, where they are positioned to directly support B cell expansion and differentiation [14], [15]. Tfh differentiation is driven by expression of the transcriptional repressor B-cell lymphoma-6(Bcl-6), which turns on a program that guides T cells close to B-cell areas [16]. Sustained Bcl-6 expression promotes the entry of Tfh cells into follicles and modulates their cytokine expression profile so they can support and select germinal center B cells that have acquired affinity-enhancing mutations in their immunoglobulin genes [12]. Tfh cells express a unique combination of effector molecules that are critical for their development and function, including high levels of the surface receptors ICOS, CD40 ligand (CD40L), PD-1, BTLA and CD84 [13], [17]. The cytokine IL-21 is critical for the formation of germinal centers and the development of Tfh cells [18].

The contribution of Tfh cells to autoimmune disease has recently received invigorated interest because of the demonstration that this lymphocyte population is important not only for generating from naïve T cells during an immune response but also for helping B cell to secrete autoantibodies. In addition, The pairing of Tfh cells and GC B cells occurs at the transcriptional level as the Bcl-6–IRF4–Blimp-1 axis, which is crucial for B cell differentiation, is also essential for the Tfh cell identity [19].The *sanroque* mouse have been instructive in highlighting the role of Tfh cells in promotion of systemic autoimmunity [17]. Recent studies have shown that circulating Tfh cells increased in some patients with infection [20] and autoimmune diseases [21]–[23]. However, little is known on the frequencies of Tfh cells in T1D patients. Moreover, it is not yet known whether the interaction between Tfh cells and B cells influences the process of T1D or not. Therefore, we sought to explore the role of circulating Tfh cells in patients with T1D.

## Materials and Methods

### Study design and patients

54 patients (<2 year from disease onset) with T1D (24 female and 30 males; mean± SEM age 23.13±13.16 years) were enrolled in the study. The diagnosis was based on the criteria of World Health Organization and American Diabetes Association. Fasting serum C-Peptide was measured by chemiluminescence (Roche Diagnostics, Basel, Switzerland). Rituximab therapy was administered intravenously at a dose of 125 mg/m^2^ surface area at weeks 0, 1, 2, 3. Peripheral blood samples were obtained from all patients and healthy controls. Blood was isolated for analyses just before infusions on weeks 0, and 16 from 20 patients with rituximab therapy. 31 age- and sex-matched healthy volunteers were recruited as controls-namely, 14 females and 17 males, ranging from 26.61±7.35 years. All of the control subjects were free of a history of T1D or autoimmune diseases. All participants provided their written consent to participate in all stages of the study. We obtained written informed consent from the guardians on the behalf of the minors/children participants. Ethical approval for the research (including the consent procedure) was granted by Human Ethics Committee of the First Affiliated Hospital of Nanjing Medical University, Jiangsu, China.

### Islet autoantibody determination

Serum autoantibodies were measured by radio-binding assays, using ^35^S labeled glutamic acid decarboxylase-65 (GAD65), protein-tyrosine-phosphatase-2 (IA-2) and zinc transporter 8 (ZnT8). As previously described [24], antibody levels were expressed as a relative immunoprecipitation index, which is defined as (sample – negative control)/(positive control - negative control). The cut-off for positivity for GADA, IA2A and ZnT8A was defined as a value above 0.015, 0.048, and 0.018 respectively, based on the 99th percentile of 102, 315 and 218 healthy control subjects (non-diabetic individuals without known autoimmune disease and no family history of diabetes).

### Flow cytometry

PBMCs at 10^6^/tube were stained in duplicate with APC-anti-CXCR5 (R&D Systems) and FITC-anti-CD4, PE-anti-CD278(eBioscience, San Diego, USA), or isotype-matched control IgG (eBioscience, San Diego, USA) at room temperature for 30 minutes, respectively. After being washed with PBS, the cells were subjected to flow cytometry analysis using a FACSCalibur (Beckton Dickinson) and analyzed by FlowJo software (v7.6.4). The cells were gated on the forward scatter of living cells and then centered on CD4^+^ T cells. Subsequently, theCD4^+^CXCR5^+^ICOS^+^ Tfh cells were determined by flow cytometric analysis, and at least 50,000 events per sample were analyzed.

### Enzyme-linked ImmunoSorbent assay (ELISA)

The concentrations of serum IL-21 and IL-6 from individual patients and healthy controls were determined using ELISA kits, according to the manufacturers' instruction (Biolegend, San Diego, CA).

### Cell Isolation

Human peripheral blood mononuclear cells (PBMC) were isolated by LymphoprepTM (Nycomed, Pharma AS, Oslo, Norway) gradients according to the manufacturer's protocol. CD4^+^ T cells were purified from PBMC by microbead-conjugated antihuman CD4 monoclonal Ab (mAb) (Miltenyi Biotec GmbH, Bergisch Gladbach, Germany) according to the manufacturer's instructions.

### RNA isolation and Real-time PCR

TRIzol reagent (Invitrogen, Carlsbad, CA) was added to CD4+ T cells. Total RNA was extracted with TRIzol reagent and cDNA synthesized according to manufacurer's instructions (Takara, Japan). Real-time PCR was performed in duplicate using SYBR Premix Ex TaqTM (Takara, Japan). Primer sequences were as follows: IL-21, sense, 5′-CACAGACTAACATGCCCTTCAT-3′; antisense, 5′-GAATCTTCACTTCCGTGTGTTCT-3′, IL-6, sense, 5′-CACACAGACAGCCACTCACC-3′; antisense, 5′-TTTTCTGCCAGTGCCTCTTT-3′, Bcl-6, sense, 5′-AAGGCCAGTGAAGCAGAGA-3′; antisense, 5′-CCGATAGGCCATGATGTCT-3′. Each gene was normalized to GAPDH with the following primers: sense 5′-AAGGTGAAGGTCGGAGTCAA-3′; antisense, 5′-TGGACTCCACGACGTACTCA-3′.

### Statistical analysis

GraphPad PRISM 5.0 Software was used for statistical analysis (GraphPad Software, Inc., San Diego, CA). Values were expressed as means ±SD according to their distribution. Student's unpaired or paired *t* test was performed over all statistically significant changes between two groups. *p*<0.05 were considered to be statistically significant. Correlations between variables were determined by Spearman's correlation coefficient.

## Results

### Increased frequencies of circulating Tfh cells in T1D patients

**Figure 1:**
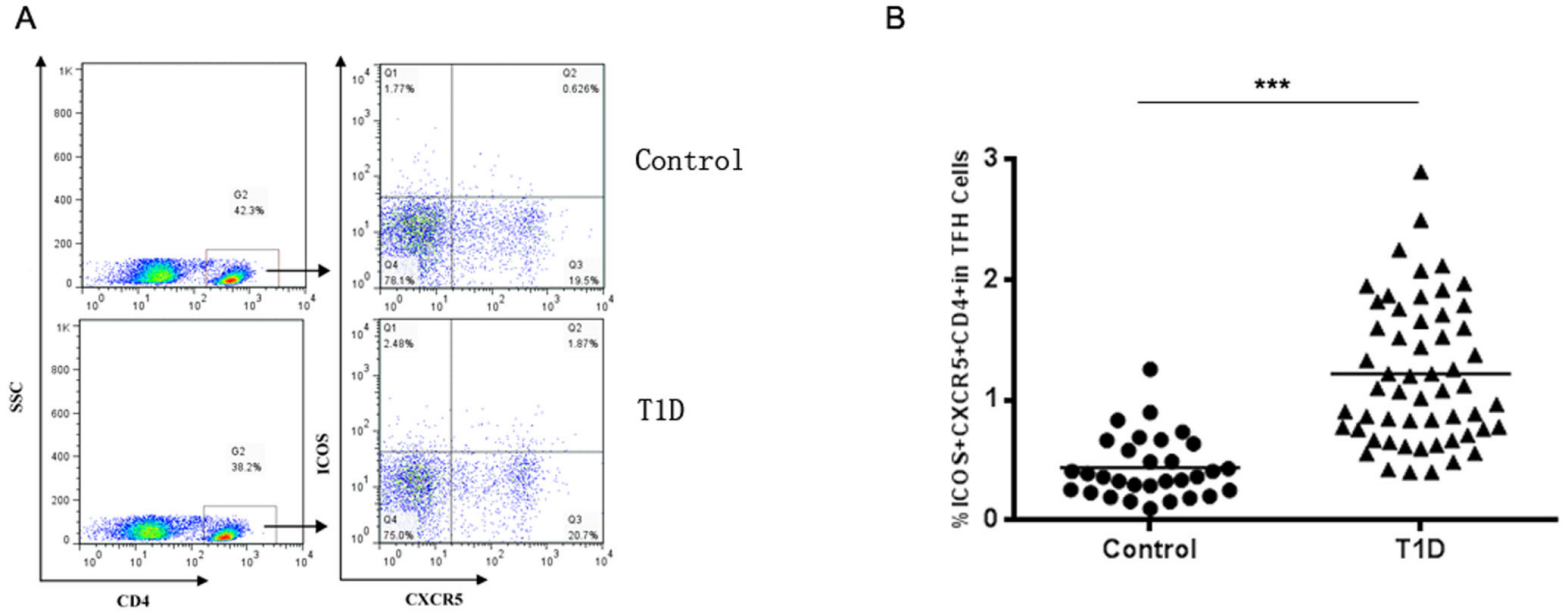
The percentages of CD4^+^CXCR5^+^ICOS5^+^ T cells in peripheral blood of patients with T1D. Peripheral blood mononuclear cells (PMBCs) from T1D patients (*n* = 54) and healthy controls (*n* = 31) were stained with labelled antibodies as described in Methods. A. Representative dot plots of CD4^+^CXCR5^+^ICOS5^+^ T cells in different groups of samples. Values in the *upper right quadrant* correspond to the percentages of CD4^+^CXCR5^+^ICOS5^+^ T cells. At least about 50,000 events were analyzed for each sample. B. CD4^+^CXCR5^+^ICOS5^+^ T cells were compared between T1D patients and healthy controls. Each *data point* represents an individual subject. The bars indicate the mean values. Student's unpaired *t* test was performed. ***, P<0.001.

To investigate the potential role of peripheral Tfh cells in T1D patients, the frequencies of peripheral blood CD4^+^CXCR5^+^ICOS^+^ T cells were analyzed by flow cytometry (Fig. 1A). The frequencies of CD4^+^CXCR5^+^ICOS^+^ T cells in PBMCs was significantly increased in T1D patients compared with healthy controls (*p*<0.0001) (Fig. 1B).

### High Levels of Autoantibodies with Increased Frequencies of the Circulating Tfh Cells in T1D Patients

**Figure 2:**
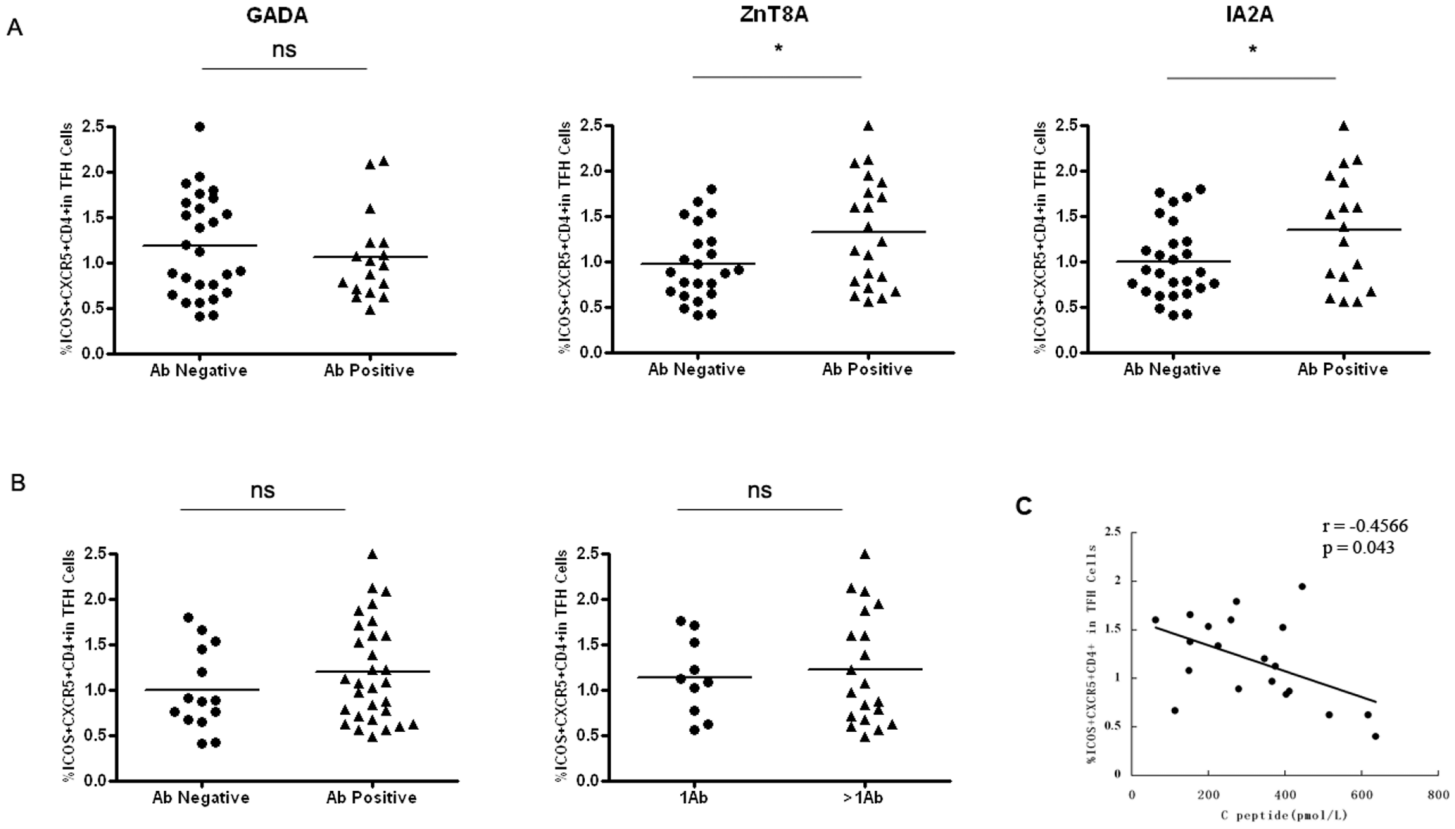
The correlation of CD4^+^CXCR5^+^ICOS5^+^ T cells expression levels with autoantibody number (n = 44) and fasting serum C-peptide in T1D patients (n = 20). A. Frequencies are shown of CD4^+^CXCR5^+^ICOS5^+^ subsets from seronegative vs. seropositive subjects. B. The percentages of CD4^+^CXCR5^+^ICOS5^+^ were stratified into two groups based on the expression of one and more than one anti-islet autoantibodies. C. The correlation between the percentages of CD4^+^CXCR5^+^ICOS5^+^ T cells in PBMCs and fasting C-peptide in 20 T1D patients. The bars indicate the mean values. Student's unpaired *t* test was performed. *, P<0.05, ns, No significant differences.

Ab responses against most antigens require interactions between B cells and CD4+ T helper cells, and Tfh specialize in providing cognate help to B cells. GADA, ZnT8A and IA2A are all critical autoantibodies which have been used for the diagnosis of T1D. In order to analyze the association between autoantibodies and the circulating Tfh cells, we compared the levels of serum autoantibodies and frequencies of the circulating CD4^+^CXCR5^+^ICOS^+^ T cells in T1D patients. According to the titer of autoantibodies, T1D patients were divided into two groups: autoantibody-positive group and autoantibody-negative group. In Fig. 2A, the percentages of circulating Tfh cells increased in autoantibody-positive subjects versus the autoantibody-negative subjects in ZnT8A (p = 0.026) and IA2A (*p* = 0.0308). In contrast, there was no significant difference in the percentages of circulating Tfh cells between T1D patients with positive GADA and negative GADA. We further evaluated whether the magnitude of the Tfh cells responsiveness is associated with the number of autoantibodies. However, we found the percentages of Tfh cells did not increase in seropositive versus seronegative subjects or in individuals with more than one autoantibody versus subjects with only one autoantibody (Fig. 2B).

More importantly, Spearman's correlation analysis revealed that the frequencies of CD4+CXCR5+ ICOS+ T cells was significantly correlated with the concentrations of fasting serum C-peptide in 20 T1D patients. (r = −0.4566, p = 0.043, Fig. 2C).

### Gene expression and cytokine concentrations in T1D patients

**Figure 3:**
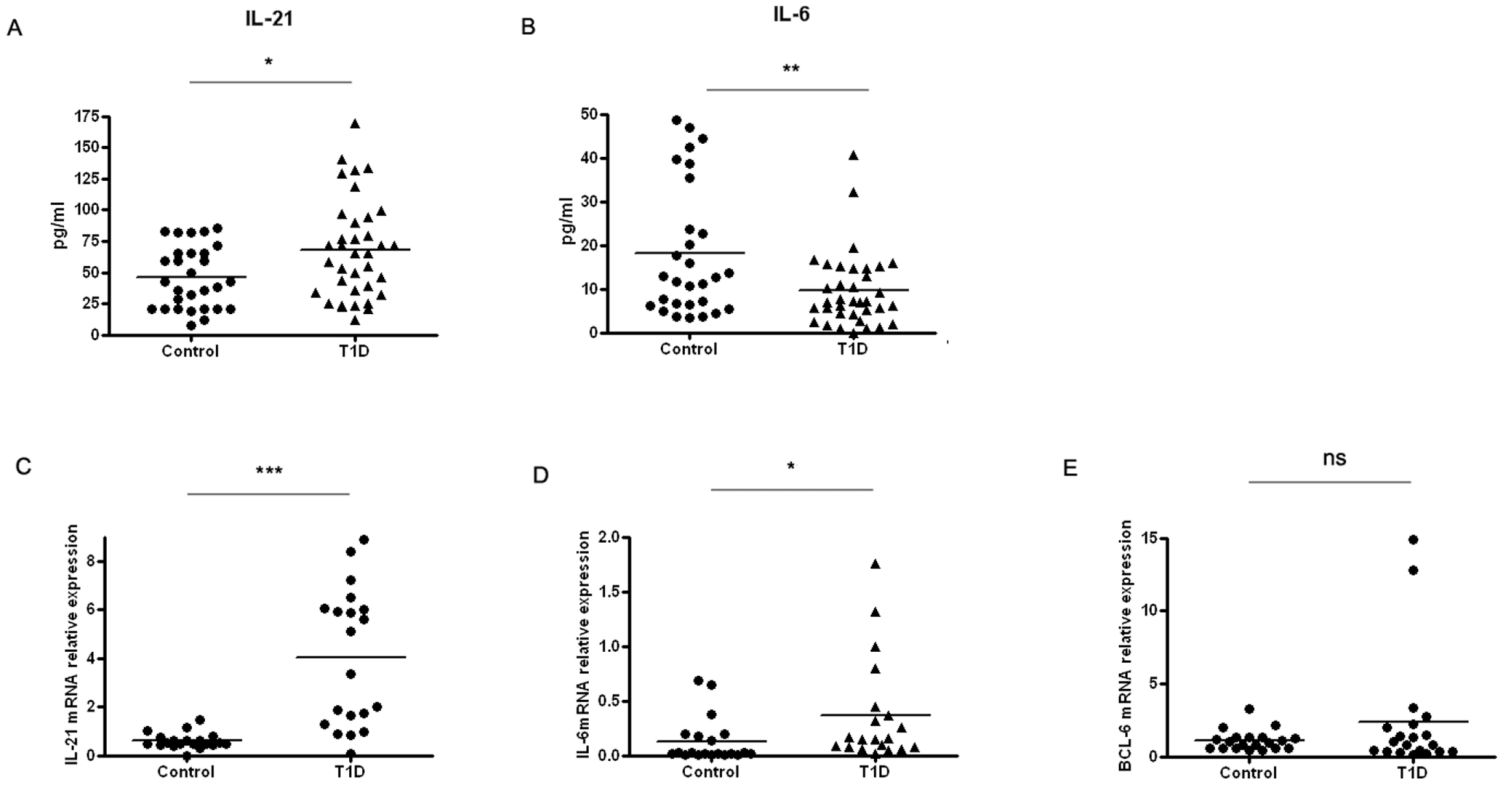
The expression of IL-21, IL-6 and Bcl-6 in T1D patients. A. The levels of serum IL-21 in T1D patients (*n* = 36 and healthy controls (*n* = 29). B. The levels of serum IL-6 inT1D patients (*n* = 36) and healthy controls (*n* = 29). C. The levels of IL-21 mRNA in CD4+ T cells were detected by real-time PCR in T1D patients (*n* = 20) and healthy controls (*n* = 20). D. The levels of IL-6 mRNA in CD4+ T cells were detected by real-time PCR in T1D patients (*n* = 20) and healthy controls (*n* = 20). E. The levels of BCL-6 mRNA in CD4+ T cells were detected by real-time PCR inT1D patients (*n* = 20) and healthy controls (*n* = 20). Student's unpaired *t* test was performed. *, P<0.05, **, P<0.01, ***, P<0.001, ns, No significant differences.

IL-21 and IL-6 have critical roles in the Tfh population. There were increased IL-21 and decreased IL-6 concentrations in sera of T1D patients compared with healthy controls (*p* = 0.0104, *p* = 0.0047)(Fig. 3A and B). The IL-21 and IL-6 mRNA expression was significantly increased in T1D patients (*p*<0.0001, P = 0.0472) (Fig. 3C and D).The Previous studies demonstrated that the Bcl-6 was a key transcription factor for Tfh cell differentiation [25]. We assessed the expression of transcription factor Bcl-6 in T1D patients and healthy controls. However, there were no significant differences for Bcl-6 mRNA between T1D and control groups (Fig. 3E).

### Treatment with rituximab reduced the circulating Tfh cells in T1D patients

**Figure 4:**
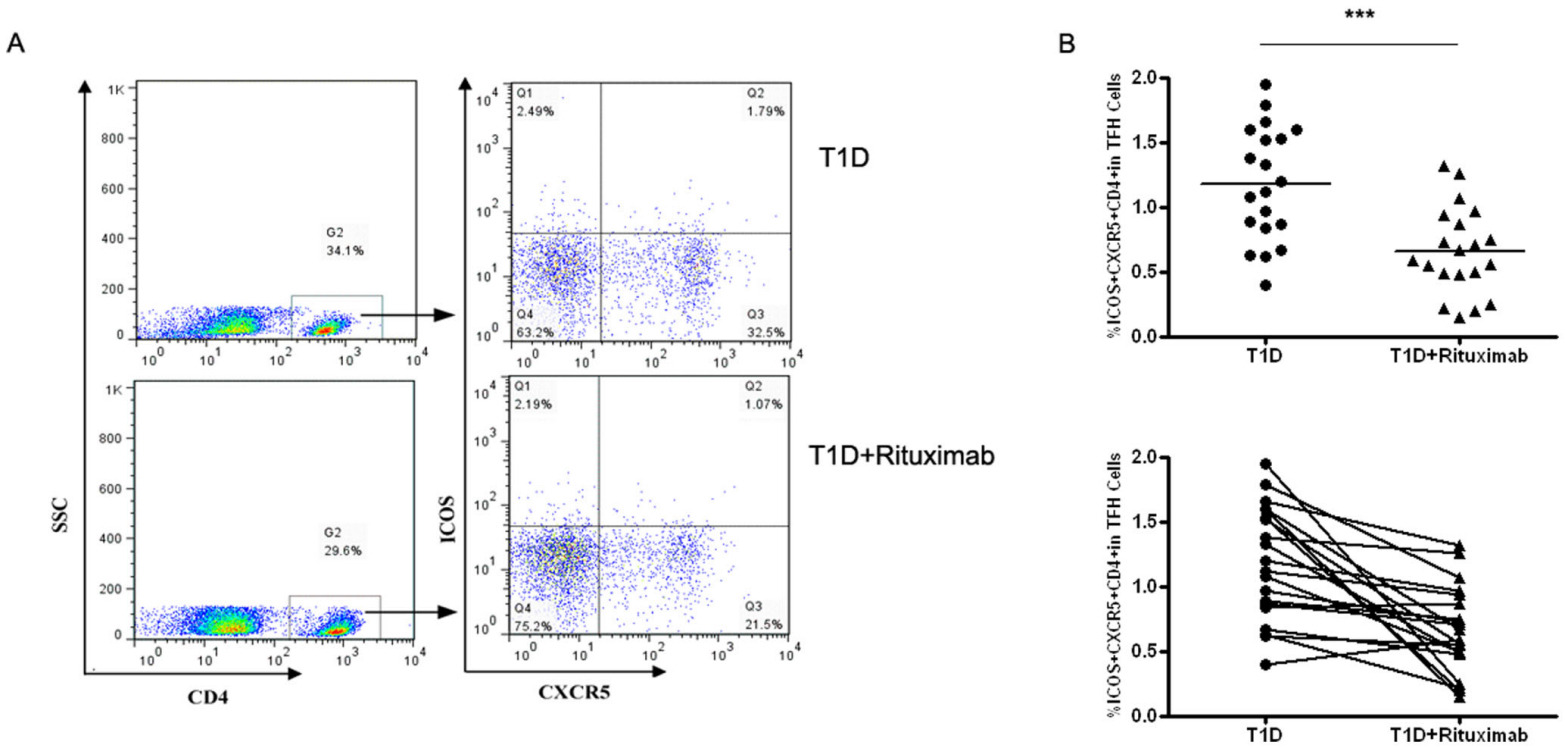
The percentages changes of CD4^+^CXCR5^+^ICOS5^+^ T cells in T1D patients between pretherapy and post-treatment groups (n = 20). A. Representative dot plots of CD4^+^CXCR5^+^ICOS5^+^ T cells in different groups of samples. Values in the *upper right quadrant* correspond to the percentages of CD4^+^CXCR5^+^ICOS5^+^ T cells. At least about 50,000 events were analyzed for each sample. B. CD4^+^CXCR5^+^ICOS5^+^ T cells were compared between pretherapy and post-treatment patients. Each *data point* represents an individual subject. The bars indicate the mean values. Student's paired *t* test was performed. ***, P<0.01.

Rituximab is an anti-CD20 mAb that is potent B cell depletion. To further demonstrate the interaction of Tfh cells and B cells in T1D patients, twenty of 54 patients evaluated in our study received rituximab intravenously. T1D patients who received rituximab treatment had efficiently depleted the CD20-positive B cells in the course of treatment (data not shown). Their frequencies of Tfh cells were characterized before and after drug treatment, respectively (Fig. 4A). Notably, following treatment with rituximab, the frequencies of Tfh cells were reduced significantly, as compared with that of before treatment (*p* = 0.0003, Fig. 4B).

### Rituximab reduces islet-related humoral responses and significantly modulated serum cytokines and mRNA expression in drug response patients

**Figure 5:**
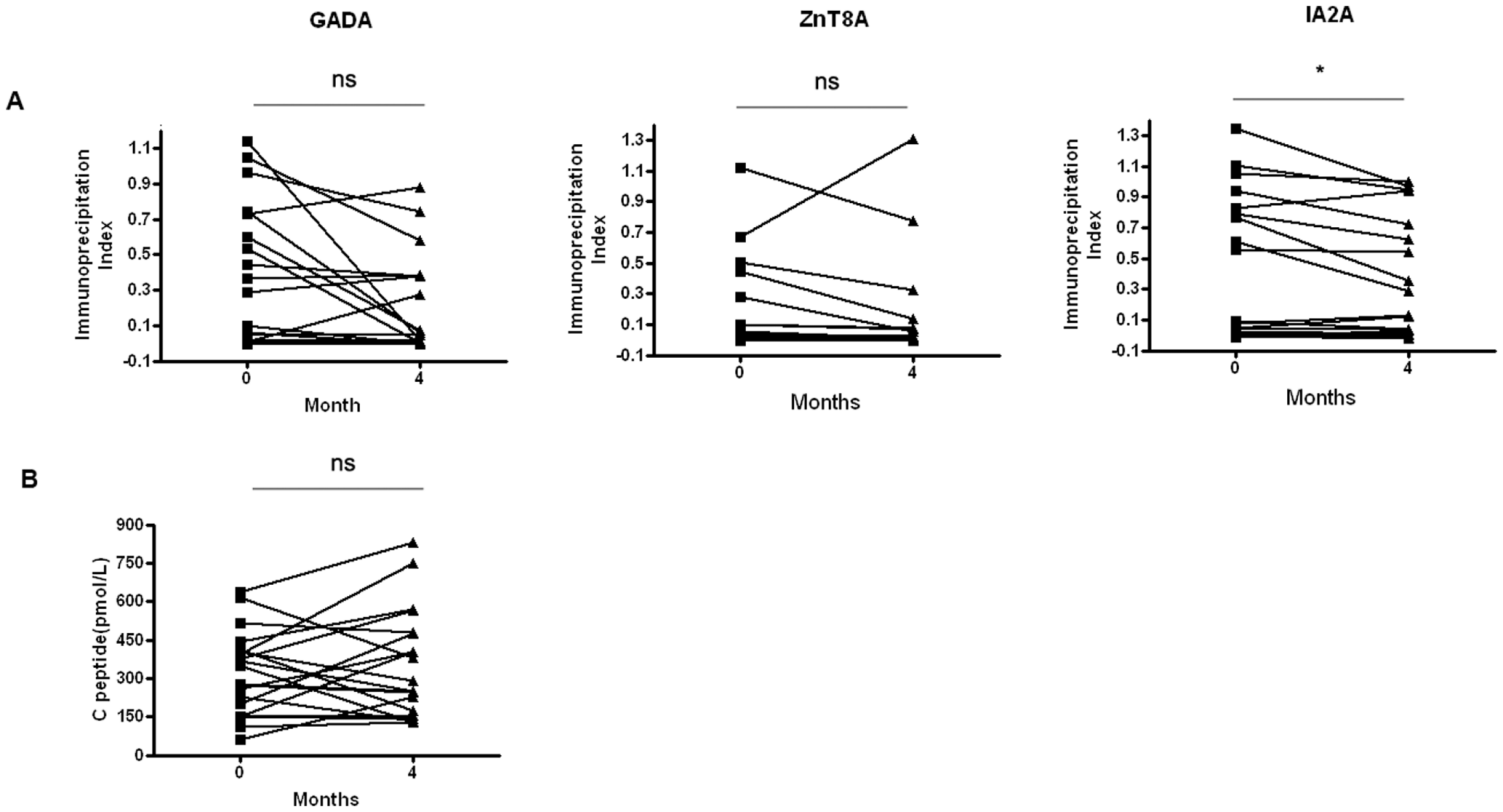
The changes of autoantibody responses and fasting C peptide in the 4 month follow-up group (n = 20). A. Serum samples obtained from T1D patients before and after treatment. Abs were measured by radio-binding assays as described in Methods. Autoantibody levels were calculated as the immunoprecipitation index. B. Initial and final of serum fasting C peptide in the 6 month follow-up group. Student's paired *t* test was performed. *, P<0.05, ns, No significant differences.

To further understand the effect of treatment with rituximab, we detected the levels of serum autoantibodies before and after drug treatment. In most individuals, IA2A titers typically declined over the course of the 4 months follow-up (*p* = 0.0309) (Fig. 5A). There was no significantly decrease with GADA and ZnT8A. Beta cell function did not show significant improvement during the therapy (Fig. 5B). However, 10 of 20 patients increased their levels of fasting serum C-peptide after 4 months therapy.

**Figure 6:**
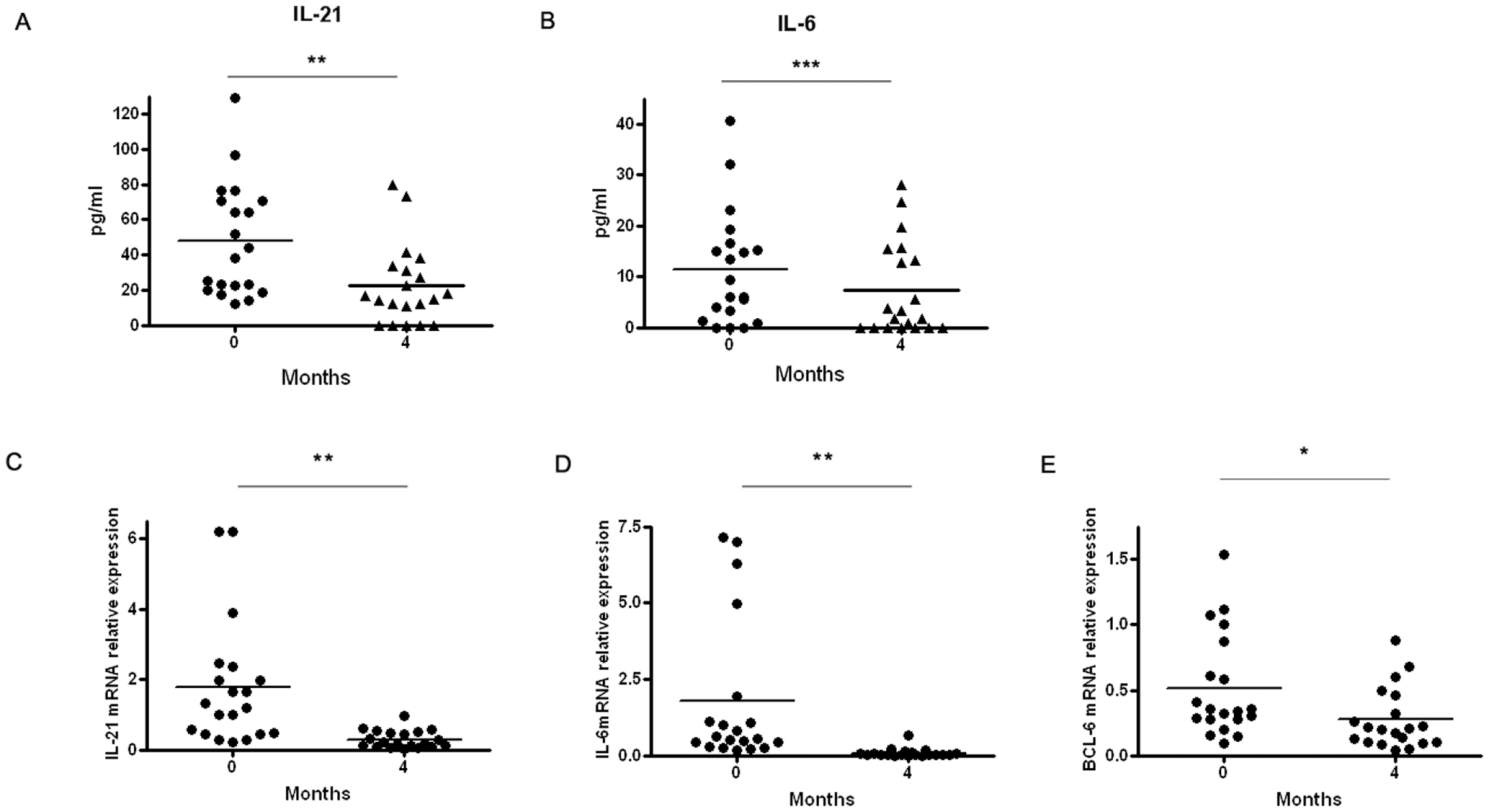
The expression of IL-21, IL-6 and Bcl-6 from T1D patients before and after treatment (n = 20). A. The levels of serum IL-21 in pretherapy and posttreatment groups. B. The levels of serum IL-6 in pretherapy and posttreatment groups. C. The levels of IL-21 mRNA in CD4+ T cells were detected by real-time PCR in pretherapy and posttreatment groups. D. The levels of IL-6 mRNA in CD4+ T cells were detected by real-time PCR in pretherapy and posttreatment groups. E. The levels of BCL-6 mRNA in CD4+ T cells were detected by real-time PCR in pretherapy and posttreatment groups. Student's paired *t* test was performed. *, P<0.05, **, P<0.01, ***, P<0.001.

IL-21 and IL-6 are produced by Tfh cells and act directly on B cells to maximize Bcl-6 expression and promote GC B cell growth [26]–[28]. We compared the serum cytokine levels of IL-21 and IL-6 from those patients who took rituximab and those who did not. We found that the concentrations of serum IL-21 and IL-6 were significantly lower after treatment in those patients (*p* = 0.0016, *p* = 0.0006) (Fig. 6A and B). At same time, real-time PCR also showed that IL-21, IL-6 and Bcl-6 expression was reduced after treatment with rituximab (*p* = 0.0019, *p* = 0.0056 and 0.0203) (Fig. 6C, D and E).

## Discussion

Tfh cells are key regulators of humoral immunity. Tfh cells have a crucial role in helping B cell maturation and the production of antibodies in response to foreign antigens [29]. The present studies also characterized the frequency of peripheral Tfh cells in several autoimmune diseases [21], [23], [30]. In the present study, we demonstrated that circulating CD4^+^CXCR5^+^ICOS^+^Tfh cells significantly increased in T1D patients. In addition, B-cell depletion therapy for 16 weeks could significantly reduce these T cell subsets. The findings clearly indicated that interaction between Tfh cells and B cells participated in T1D-related immune responses.

B cells have multiple roles in the development and organization of the immune system and play key, albeit ill-defined, roles in driving cell autoimmunity. Several studies indicate that B cells can serve as APCs or prime cell-specific T cells. Consequently, targeting B cells offers a potential approach to modulate beta cell-specific autoimmunity. Tfh cells are the class of effector T helper cells that regulates the step-wise development of antigen-specific B cell immunity in vivo [31]. The function of Tfh cells is an antigen-bridge for T–B co-operation helped to establish the basic tenets of ‘cognate’ help for antigen-specific B cell immunity. B-cell depletion therapy further suggested the interaction of Tfh cells and B cells in T1D patients. We did not observe significant correlations between autoantibody levels and frequencies of circulating Tfh. However, we found that Tfh cells had significant differences between the autoantibody-positive group and negative group in ZnT8A and IA2A. Our results also showed that variation of Tfh cells in diabetic patients correlated with C-peptide. Interestingly, only the titers of IA2A declined following B cell depletion, suggesting that some autoantibody responses are continuously initiated by cohorts of CD20-expressing B cells whereas others are probably maintained by long-lived CD20-negative plasma cells [32]. Regardless of this, many studies found that B cell depletion was clinically effective even in patients that did not show decreased autoantibody titers [33], [34]. It was indicated that B cells must also contribute to pathology by mechanisms other than autoantibody production. There was no significant difference in C-peptide during the treatment. It may be due to the short observation period or not large enough samples with rituximab therapy. However, ten of 20 patients increased their levels of fasting serum C-peptide after 4 months therapy. It was suggested that B and Tfh cell collaboration could participate in the pathogenesis of T1D. B cells seemed to enhance autoreactive Tfh cell responses in T1D patients.

Tfh cells, critical helpers of B cells and drivers of autoimmune disease via GCs are major producers of IL- 21 [18]. Tfh and NKTfh cells produce IL-21 to enhance B cell differentiation toward both extrafollicular and GC pathways [35]. Within GCs, IL-21 signals directly to GC B cells to maximize Bcl-6 expression and sustain the GCs [27]. Our data was consistent with other evidence that the level of serum IL-21 was increased in T1D. Furthermore, analysis of the IL-21mRNA expression in circulating CD4+ T cells yielded a higher expression. Whereas, this population is clearly reduced after B-cell depletion, which indicated that B cells may reflect the level of cytokine IL-21. Conflicting data regarding IL-6 serum levels in autoimmune forms of diabetes have been reported. Some groups reported lower levels of serum IL-6 in type 1 diabetes [36]–[38] while others have found normal or even increased levels of IL-6. However, in our study IL-6 levels in T1D patients did not reach the levels observed in healthy children. It is possible that IL-6 rise from their lowest values in newly diagnosed cases to posttreatment values, and increase further in T1D patients monitored over a long term [38]. Bcl-6 is required for a T and B cell antigen-specific extrafollicular antibody response. However, analysis of the Bcl-6 mRNA expression in circulating CD4+ T cells yielded no differences in patients with T1D, which is consistent with previous studies [23], [30]

Tfh cells select mutated B cells in GCs and targets immunoglobulin (Ig) variable region genes of rapidly dividing germinal centre B cells [39]. This can lead to an increase in the affinity of the B-cell receptor for the immunizing antigen, but there is abundant evidence that this stochastic process can also generate self-reactive specificities [39]. Furthermore, once self-reactive B cells have been vaguely selected in GCs, their differentiated offspring can live and produce antibodies unchecked, subject to virtually no further control. Consequently, autoimmune disease may occur. A tightly controlled process of germinal centre B-cell selection by antigen-specific Tfh cells is normally in place to ensure positive selection of those cells with the highest affinity towards foreign antigens while preventing selection of cells that have become self-reactive. We found that the frequency of Tfh cells increased significantly in the peripheral blood of T1D patients. It was significant correlation between Tfh cells and C-peptide before therapy. Follow-up analysis showed that Tfh cells decreased in 19 out of 20 patients who treated with Rituximab and 10 out of these 10 patients who showed improved fasting C-peptide levels after therapy. It indicated that Tfh cells might participate in the T1D-relatede immune responses. Several studies found that antigen-specific Tfh cells have been identified in the peripheral blood of humans [40]. Streeck H et al. reported that HIV-specific Tfh cell populations were significantly expanded in chronic HIV infection and were highly associated with viremia [41]. Tfh cells have emerged as being critical to prevent the development of diseases. Thus, antigen-specific Tfh cells may be pathogenic in T1D.

Our data preliminarily indicated that circling CD4^+^CXCR5^+^ICOS^+^Tfh cells could be involved in T1D. Furthermore, we suggested interaction of Tfh cells and B cells in T1D patients by rituximab treatment. However, the detailed characterization of the Tfh cells and its pathogenic process in T1D will be a challenge for the future.
